# Supplementary material for: Use of Mass Spectrometry to Determine the Diversity of Toxins Produced by Gambierdiscus and Fukuyoa Species from Balearic Islands and Crete (Mediterranean Sea) and the Canary Islands (Northeast Atlantic)
Source: Toxins (Basel). 2020 May 7;12(5):305. doi: 10.3390/toxins12050305 (PMC7291038; doi:10.3390/toxins12050305)
Supplement: Supplementary file 1 [file toxins-12-00305-s001.pdf]

Supplementary material for: Use of Mass Spectrometry to Determine the Diversity of Toxins Produced by *Gambierdiscus* and *Fukuyoa* Species from Balearic Islands and Crete (Mediterranean Sea) and the Canary Islands (Northeast Atlantic)

Pablo Estevez, Manoëlla Sibat, José Manuel Leão-Martins, Angels Tudó, Maria Rambla-Alegre, Katerina Aligizaki, Jorge Diogène, Ana Gago-Martinez and Philipp Hess

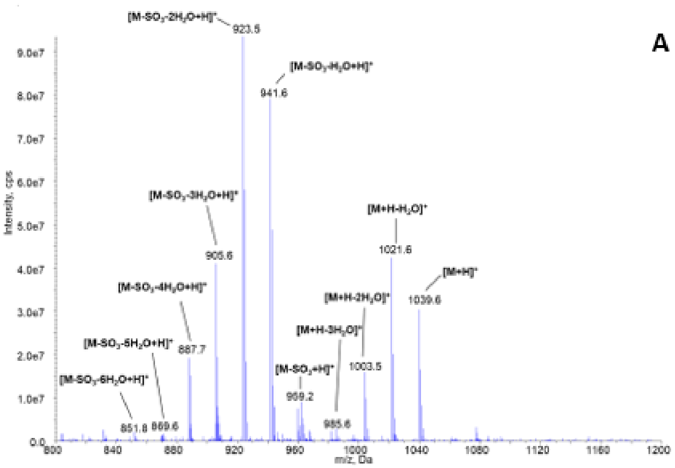

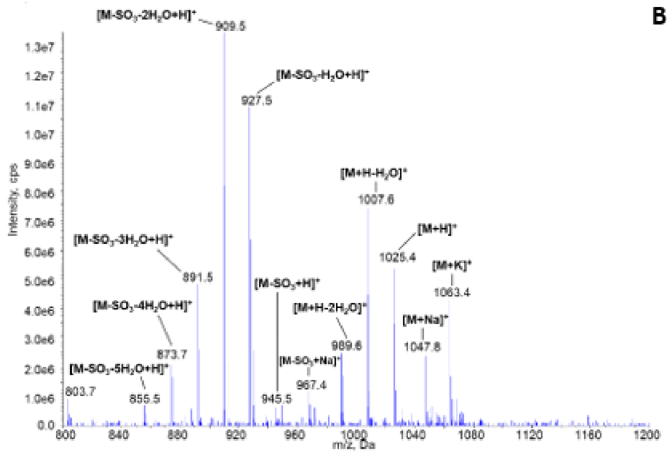

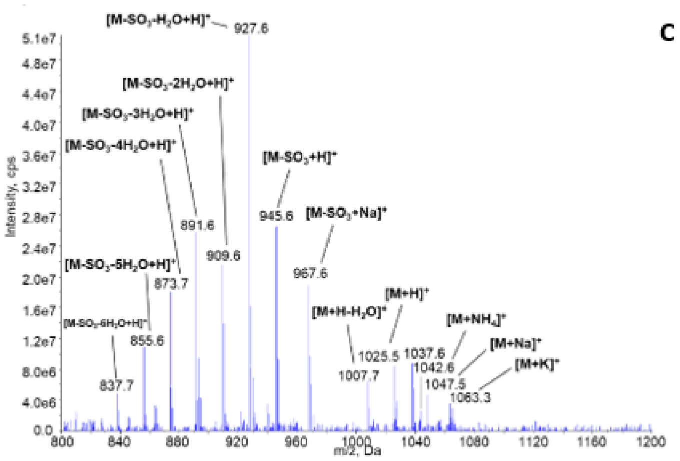

Figure S1. LC-MS/MS Full scan analysis of: (**A**) MTX3 from *G. australes*; (**B**) gambierone from *Gambierdiscus sp;* (**C**) putative gambierone analogue from *G. australes*.

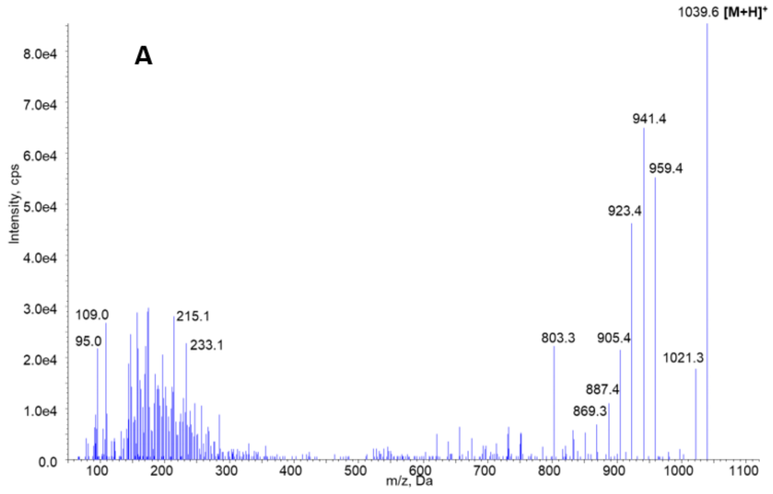

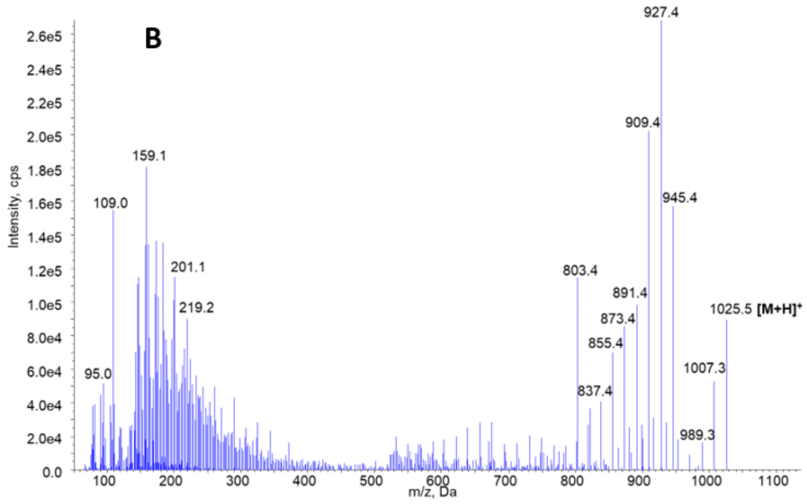

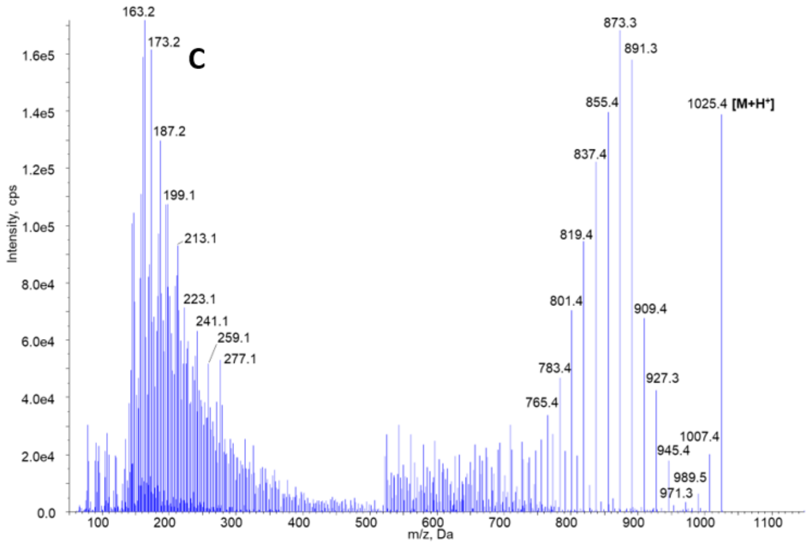

Figure S2. LC-MS/MS spectra resulting from enhanced product ion scan at an average CE of 20, 40 and 60 eV of: (**A**) MTX3 from *G. australes*; (**B**) gambierone from *Gambierdiscus sp;* (**C**) putative gambierone analogue from *G. australes.*

Table S1. Accurate mass measurements using LC-HRMS full scan analysis in ESI⁻ and ESI⁺ mode for MTX3.

Specie	Sample ID	Injection N°	ESI⁺ MTX3 Ion $[M+H-H_2O]^+$	$[M+H]^+$	$[M+NH_4]^+$	$[M+Na]^+$	Retention time (min)	ESI⁻ MTX3 Ion $[M-H]^-$	Retention time (min)
G. australes	IRTA-SMN-17-189	R1	1021.4835 (Δppm = +1.0)	1039.4957 (Δppm = +2.5)	1056.5207 (Δppm = +1.0)	1061.4730 (Δppm = −1.9)	7.67	1037.4789 (Δppm = +0.4)	7.66
		R2	1021.4832 (Δppm = +0.7)	1039.4956 (Δppm = +2.4)	1056.5196 (Δppm = +0.0)	1061.4734 (Δppm = −1.5)	7.66	1037.4791 (Δppm = +0.6)	7.66
		R3	1021.4835 (Δppm = +1.0)	1039.4959 (Δppm = +2.7)	1056.5207 (Δppm = +1.0)	1061.4739 (Δppm = −1.0)	7.66	1037.4791 (Δppm = +0.6)	7.66
	IRTA-SMN-17-253	R1	1021.4840 (Δppm = +1.5)	1039.4963 (Δppm = +3.1)	1056.5211 (Δppm = +1.4)	1061.4744 (Δppm = −0.6)	7.63	1037.4785 (Δppm = +0.0)	7.63
		R2	1021.4845 (Δppm = +2.0)	1039.4969 (Δppm = +3.7)	1056.5220 (Δppm = +2.3)	1061.4741 (Δppm = −0.8)	7.61	1037.4791 (Δppm = +0.6)	7.61
		R3	1021.4843 (Δppm = +1.8)	1039.4966 (Δppm = +3.4)	1056.5214 (Δppm = +1.7)	1061.4739 (Δppm = −1.0)	7.61	1037.4792 (Δppm = +0.7)	7.63
	IRTA-SMN-17-244	R1	1021.4841 (Δppm = +1.6)	1039.4966 (Δppm = +3.4)	1056.5213 (Δppm = +1.6)	1061.4740 (Δppm = −0.9)	7.67	1037.4788 (Δppm = +0.3)	7.63
		R2	1021.4843 (Δppm = +1.8)	1039.4964 (Δppm = +3.2)	1056.5212 (Δppm = +1.5)	1061.4746 (Δppm = −0.4)	7.67	1037.4793 (Δppm = +0.8)	7.64
		R3	1021.4842 (Δppm = +1.7)	1039.4966 (Δppm = +3.4)	1056.5212 (Δppm = +1.5)	1061.4744 (Δppm = −0.6)	7.65	1037.4791 (Δppm= +0.6)	7.64
	IRTA-SMN-17-162	R1	1021.4839 (Δppm = +1.4)	1039.4962 (Δppm = +3.0)	1056.5210 (Δppm = +1.3)	1061.4736 (Δppm = −1.3)	7.62	1037.4788 (Δppm = +0.3)	7.66
		R2	1021.4840 (Δppm = +1.5)	1039.4962 (Δppm= +3.0)	1056.5212 (Δppm = +1.5)	1061.4737 (Δppm = −1.2)	7.66	1037.4787 (Δppm = +0.2)	7.66
		R3	1021.4838 (Δppm = +1.3)	1039.4962 (Δppm = +3.0)	1056.5213 (Δppm = +1.6)	1061.4737 (Δppm = −1.2)	7.65	1037.4790 (Δppm = +0.5)	7.65
	IRTA-SMN-17-164	R1	1021.4841 (Δppm = +1.6)	1039.4964 (Δppm = +3.2)	1056.5214 (Δppm = +1.7)	1061.4742 (Δppm = −0.8)	7.67	1037.4790 (Δppm = +0.5)	7.65
		R2	1021.4843 (Δppm = +1.8)	1039.4965 (Δppm = +3.3)	1056.5215 (Δppm = +1.8)	1061.4741 (Δppm = −0.8)	7.66	1037.4791 (Δppm = +0.6)	7.65
		R3	1021.4837 (Δppm = +1.2)	1039.4961 (Δppm = +2.9)	1056.5209 (Δppm = +1.2)	1061.4741 (Δppm = −0.8)	7.65	1037.4790 (Δppm = +0.5)	7.64
	IRTA-SMN-17-271	R1	1021.4839 (Δppm = +1.4)	1039.4962 (Δppm = +3.0)	1056.5213 (Δppm = +1.6)	1061.4723 (Δppm = −2.5)	7.62	1037.4790 (Δppm = +0.5)	7.62

		R2	1021.4844 (Δppm = +1.9)	1039.4966 (Δppm = +3.4)	1056.5215 (Δppm = +1.8)	1061.4730 (Δppm = −1.9)	7.62	1037.4792 (Δppm = +0.7)	7.62
		R3	1021.4840 (Δppm= +1.5)	1039.4962 (Δppm= +3.0)	1056.5214 (Δppm= +1.7)	1061.4729 (Δppm= -2.0)	7.62	1037.4790 (Δppm= +0.5)	7.62
G. excentricus	IRTA-SMN-17-407	R1	n.d.	n.d.	n.d.	n.d.	n.d.	n.d.	n.d.
		R2	n.d.	n.d.	n.d.	n.d.	n.d.	n.d.	n.d.
		R3	n.d.	n.d.	n.d.	n.d.	n.d.	n.d.	n.d.
F. paulensis	IRTA-SMN-17-209	R1	n.d.	n.d.	n.d.	n.d.	n.d.	1037.4769 (Δppm = −1.5)	7.67
		R2	1021.4907 (Δppm = +8.0)	1039.4931 (Δppm = +0.0)	n.d.	n.d.	7.68	1037.4769 (Δppm = −1.5)	7.65
		R3	1021.4781 (Δppm = −4.3)	1039.4908 (Δppm = −2.2)	n.d.	n.d.	7.66	1037.4765 (Δppm = −1.9)	7.65
G. sp 2	0010G-CR-CCAUTH	R1	1021.4819 (Δppm = −0.6)	1039.4925 (Δppm = −0.6)	n.d.	n.d.	7.65	1037.4768 (Δppm = −1.6)	7.64
		R2	n.d.	n.d.	n.d.	n.d.	n.d.	1037.4771 (Δppm = −1.3)	7.65
		R3	n.d.	n.d.	n.d.	n.d.	n.d.	1037.4769 (Δppm = −1.5)	7.64

Table S2. Accurate mass measurements using LC-HRMS full scan analysis in ESI⁻ and ESI⁺ mode for gambierone.

Specie	Sample ID	Injection N°	ESI⁺ Gambierone Ion $[M+H-H_2O]^+$	$[M+H]^+$	$[M+NH_4]^+$	$[M+Na]^+$	Retention time (min)	ESI⁻ Gambierone Ion $[M-H]^-$	Retention time (min)
G. australes	IRTA-SMN-17-189	R1	n.d.	n.d.	n.d.	n.d.	n.d.	n.d.	n.d.
		R2	n.d.	n.d.	n.d.	n.d.	n.d.	n.d.	n.d.
		R3	n.d.	n.d.	n.d.	n.d.	n.d.	n.d.	n.d.
	IRTA-SMN-17-253	R1	n.d.	n.d.	n.d.	n.d.	n.d.	n.d.	n.d.
		R2	n.d.	n.d.	n.d.	n.d.	n.d.	n.d.	n.d.
		R3	n.d.	n.d.	n.d.	n.d.	n.d.	n.d.	n.d.
	IRTA-SMN-17-244	R1	n.d.	n.d.	n.d.	n.d.	n.d.	n.d.	n.d.
		R2	n.d.	n.d.	n.d.	n.d.	n.d.	n.d.	n.d.
		R3	n.d.	n.d.	n.d.	n.d.	n.d.	n.d.	n.d.
	IRTA-SMN-17-162	R1	n.d.	n.d.	n.d.	n.d.	n.d.	n.d.	n.d.
		R2	n.d.	n.d.	n.d.	n.d.	n.d.	n.d.	n.d.
		R3	n.d.	n.d.	n.d.	n.d.	n.d.	n.d.	n.d.
	IRTA-SMN-17-164	R1	n.d.	n.d.	n.d.	n.d.	n.d.	n.d.	n.d.
		R2	n.d.	n.d.	n.d.	n.d.	n.d.	n.d.	n.d.
		R3	n.d.	n.d.	n.d.	n.d.	n.d.	n.d.	n.d.
	IRTA-SMN-17-271	R1	n.d.	n.d.	n.d.	n.d.	n.d.	n.d.	n.d.
		R2	n.d.	n.d.	n.d.	n.d.	n.d.	n.d.	n.d.
		R3	n.d.	n.d.	n.d.	n.d.	n.d.	n.d.	n.d.
G. excentricus	IRTA-SMN-17-407	R1	n.d.	n.d.	n.d.	n.d.	n.d.	n.d.	n.d.
		R2	n.d.	n.d.	n.d.	n.d.	n.d.	n.d.	n.d.
		R3	n.d.	n.d.	n.d.	n.d.	n.d.	n.d.	n.d.
F. paulensis	IRTA-SMN-17-209	R1	n.d.	n.d.	n.d.	n.d.	n.d.	n.d.	n.d.
		R2	n.d.	n.d.	n.d.	n.d.	n.d.	n.d.	n.d.
		R3	n.d.	n.d.	n.d.	n.d.	n.d.	n.d.	n.d.
G. sp 2	0010G-CR-CCAUTH	R1	1007.4687 (Δppm = +1.8)	1025.4809 (Δppm = +3.4)	1042.5059 (Δppm = +1.8)	1047.4582 (Δppm = −1.1)	7.34	1023.4634 (Δppm = +0.5)	7.37
		R2	1007.4695 (Δppm = +2.6)	1025.4815 (Δppm= +4.0)	1042.5066 (Δppm = +2.5)	1047.4589 (Δppm = −0.5)	7.39	1023.4635 (Δppm = +0.6)	7.37
		R3	1007.4687 (Δppm = +1.8)	1025.4810 (Δppm = +3.5)	1042.5060 (Δppm = +1.9)	1047.4580 (Δppm = −1.3)	7.39	1023.4630 (Δppm = +0.1)	7.37

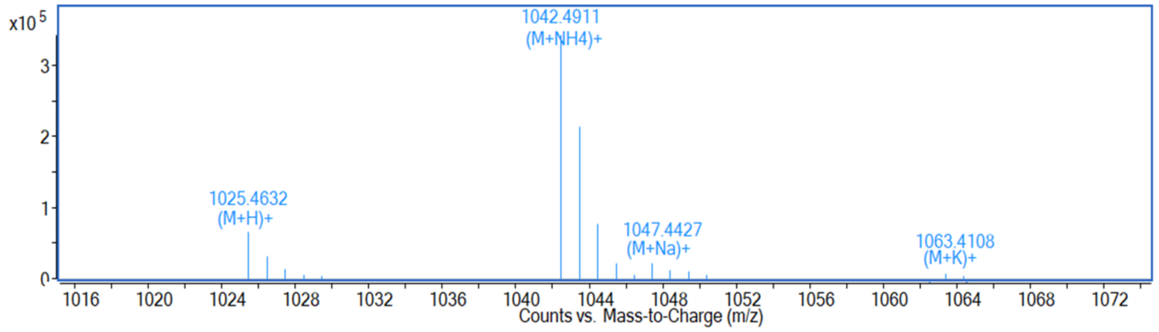

Figure S3. Putative gambierone analogue detected in LC-HRMS using the Find by Molecular Feature (FMF) algorithm in *G. australes* at 6.08 min.

Table S3. *m/z* measured values for the putative gambierone analogue and Δ ppm calculated in base of gambierone theoretical values.

**Ion**	***m/z*** **Measured**	**Theoretical**	**Δ ppm**
$[M+H]^+$	1025.4632	1025.4774	−13.8
$[M+NH_4]^+$	1042.4911	1042.5040	−12.4
$[M+Na]^+$	1047.4427	1047.4594	−15.9
$[M+K]^+$	1063.4108	1063.4333	−21.2

**Ion**	***m/z*** **Measured**	**Theoretical**	**Δ ppm**
$[M-H]^-$	1023.4449	1023.4629	−17.6
$[HOSO_3]^-$	96.9610	96.9601	+9.3

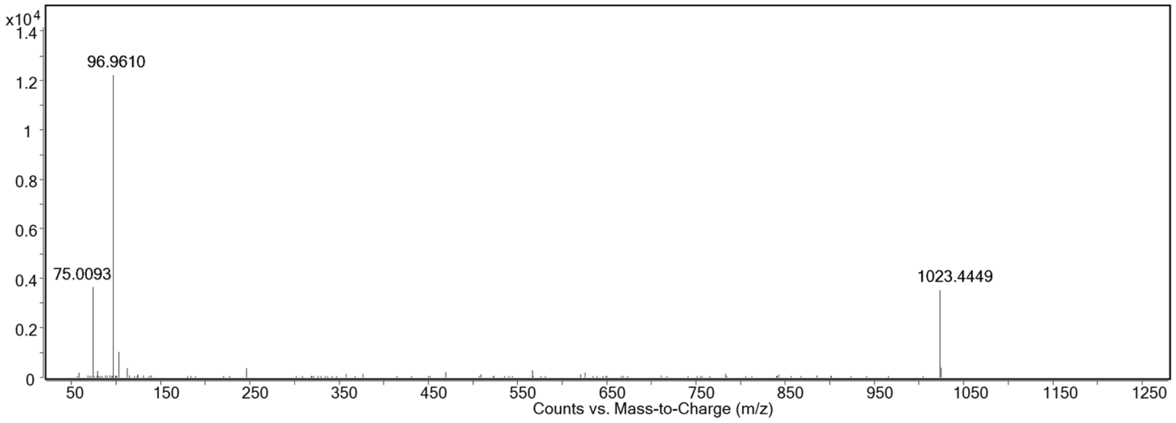

Figure S4. ESI^- Targeted MS/MS analysis of the putative gambierone analogue selecting $[M-H]^-$ ion in *G. australes* at 6.08 min. *m/z* measured values for the putative gambierone analogue and Δ ppm calculated in base of gambierone theoretical values.

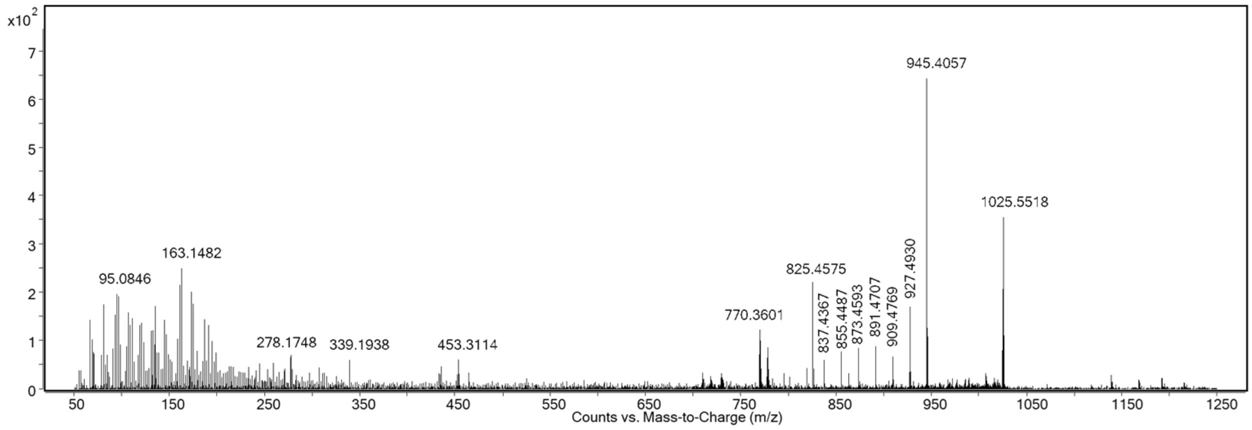

Figure S5. ESI+ Targeted MS/MS analysis of the putative gambierone analogue selecting [M+H]+ ion in *G. australes* at 6.08 min.

Table S4. Accurate mass measurements for the putative gambierone analogue and Δ ppm calculated in base of gambierone theoretical values.

**Ion**	***m/z*** **Measured** p-Gambierone	**Theoretical** Gambierone	**Δ ppm**
$[M+H]^+$	1025.5518	1025.4774	+72.6
$[M+H-H_2O]^+$	n.d.	1007.4668	n.d.
$[M+H-2H_2O]^+$	n.d.	989.4563	n.d.
$[M-SO_3+H]^+$	945.4057	945.5206	−121.5
$[M-SO_3-H_2O+H]^+$	927.4930	927.5100	−18.4
$[M-SO_3-2H_2O+H]^+$	909.4769	909.4995	−24.8
$[M-SO_3-3H_2O+H]^+$	891.4707	891.4889	−20.4
$[M-SO_3-4H_2O+H]^+$	873.4593	873.4783	−21.8
$[M-SO_3-5H_2O+H]^+$	855.4487	855.4678	−22.3
$[M-SO_3-6H_2O+H]^+$	837.4367	837.4572	−24.5

Table S5. Accurate mass measurements using LC-HRMS full scan analysis in ESI⁻ and ESI⁺ mode for putative gambieroxide.

Specie	Sample ID	Injection N°	ESI⁺ p-gambieroxide Ion $[M+H-H_2O]^+$	$[M+H]^+$	$[M+NH_4]^+$	$[M+Na]^+$	$[M+K]^+$	Retention time (min)	ESI⁻ p-gambieroxide Ion $[M-H]^-$	Retention time (min)
G. australes	IRTA-SMN-17-189	R1	n.d.	n.d.	1212.6013 (Δppm = +2.5)	1217.5541 (Δppm = +0.3)	1233.5200 (Δppm = −6.2)	5.32	1193.5582 (Δppm = +0.8)	5.32
		R2	n.d.	n.d.	1212.6009 (Δppm = +2.1)	1217.5554 (Δppm = +1.4)	1233.5098 (Δppm = −14.4)	5.32	1193.5590 (Δppm = +1.5)	5.32
		R3	n.d.	n.d.	1212.6004 (Δppm = +1.7)	1217.5530 (Δppm = -0.6)	1233.5375 (Δppm = +8.0)	5.32	1193.5587 (Δppm = +1.3)	5.31
	IRTA-SMN-17-253	R1	1177.5674 (Δppm = +5.3)	n.d.	1212.6041 (Δppm = +4.8)	1217.5577 (Δppm = +3.3)	1233.5290 (Δppm = +1.1)	5.31	1193.5603 (Δppm = +2.6)	5.31
		R2	1177.5738 (Δppm = +10.7)	n.d.	1212.6037 (Δppm = +4.5)	1217.5579 (Δppm = +3.4)	1233.5280 (Δppm = +0.3)	5.31	1193.5600 (Δppm = +2.3)	5.31
		R3	1177.5616 (Δppm = +0.3)	n.d.	1212.6034 (Δppm = +4.2)	1217.5567 (Δppm = +2.5)	1233.5193 (Δppm = −6.7)	5.31	1193.5600 (Δppm = +2.3)	5.31
	IRTA-SMN-17-244	R1	1177.5698 (Δppm = +7.3)	n.d.	1212.6036 (Δppm = +4.4)	1217.5568 (Δppm = +2.5)	1233.5313 (Δppm = +3.0)	5.30	1193.5593 (Δppm = +1.8)	5.32
		R2	1177.5616 (Δppm = +0.3)	n.d.	1212.6038 (Δppm = +4.5)	1217.5567 (Δppm = +2.5)	1233.5211 (Δppm = −5.3)	5.32	1193.5598 (Δppm = +2.2)	5.32
		R3	1177.5651 (Δppm = +3.3)	n.d.	1212.6037 (Δppm = +4.5)	1217.5570 (Δppm = +2.7)	1233.5285 (Δppm = +0.7)	5.32	1193.5598 (Δppm = +2.2)	5.32
	IRTA-SMN-17-162	R1	1177.5419 (Δppm = −16.4)	1195.5886 (Δppm = +14.1)	1212.6021 (Δppm = +3.1)	1217.5561 (Δppm = +2.0)	1233.5187 (Δppm = −7.2)	5.32	1193.5589 (Δppm = +1.4)	5.32
		R2	1177.5402 (Δppm = −17.8)	n.d.	1212.6021 (Δppm = +3.1)	1217.5559 (Δppm = +1.8)	1233.5206 (Δppm = −5.7)	5.32	1193.5593 (Δppm = +1.8)	5.32
		R3	1177.5454 (Δppm = −13.4)	1195.5818 (Δppm= +8.4)	1212.6020 (Δppm = +3.1)	1217.5568 (Δppm = +2.5)	1233.5254 (Δppm = −1.8)	5.32	1193.5594 (Δppm = +1.8)	5.32
	IRTA-SMN-17-164	R1	1177.5604 (Δppm = −0.7)	n.d.	1212.6019 (Δppm = +3.0)	1217.5553 (Δppm = +1.3)	1233.5263 (Δppm = −1.1)	5.31	1193.5591 (Δppm = +1.6)	5.32
		R2	1177.5645 (Δppm = +2.8)	n.d.	1212.6020 (Δppm = +3.1)	1217.5554 (Δppm = +1.4)	1233.5246 (Δppm = −2.4)	5.31	1193.5595 (Δppm = +1.9)	5.32
		R3	1177.5644 (Δppm = +2.7)	n.d.	1212.5983 (Δppm = 0.0)	1217.5537 (Δppm = 0.0)	1233.5210 (Δppm = −5.4)	5.31	1193.5592 (Δppm = +1.7)	5.30

	IRTA-SMN-17-271	R1	1177.5436 (Δppm = −14.9)	n.d.	1211.6027 (Δppm = +3.6)	1217.5567 (Δppm = +2.5)	1233.5231 (Δppm = −3.6)	5.32	1193.5602 (Δppm = +2.5)	5.32
		R2	1177.5450 (Δppm = −13.8)	n.d.	1212.6037 (Δppm = +4.5)	1217.5537 (Δppm = 0.0)	1233.5252 (Δppm = −1.9)	5.32	1193.5598 (Δppm = +2.2)	5.32
		R3	1177.5430 (Δppm = −15.5)	1195.5836 (Δppm = +10.0)	1212.6037 (Δppm = +4.5)	1217.5569 (Δppm = +2.6)	1233.5212 (Δppm = −5.2)	5.31	1119.5601 (Δppm = +2.4)	5.32
G. excentricus	IRTA-SMN-17-407	R1	n.d.	n.d.	n.d.	n.d.	n.d.	n.d.	n.d.	n.d.
		R2	n.d.	n.d.	n.d.	n.d.	n.d.	n.d.	n.d.	n.d.
		R3	n.d.	n.d.	n.d.	n.d.	n.d.	n.d.	n.d.	n.d.
F. paulensis	IRTA-SMN-17-209	R1	n.d.	n.d.	n.d.	n.d.	n.d.	n.d.	n.d.	n.d.
		R2	n.d.	n.d.	n.d.	n.d.	n.d.	n.d.	n.d.	n.d.
		R3	n.d.	n.d.	n.d.	n.d.	n.d.	n.d.	n.d.	n.d.
G. sp 2	0010G-CR-CCAUTH	R1	n.d.	n.d.	n.d.	n.d.	n.d.	n.d.	n.d.	n.d.
		R2	n.d.	n.d.	n.d.	n.d.	n.d.	n.d.	n.d.	n.d.
		R3	n.d.	n.d.	n.d.	n.d.	n.d.	n.d.	n.d.	n.d.

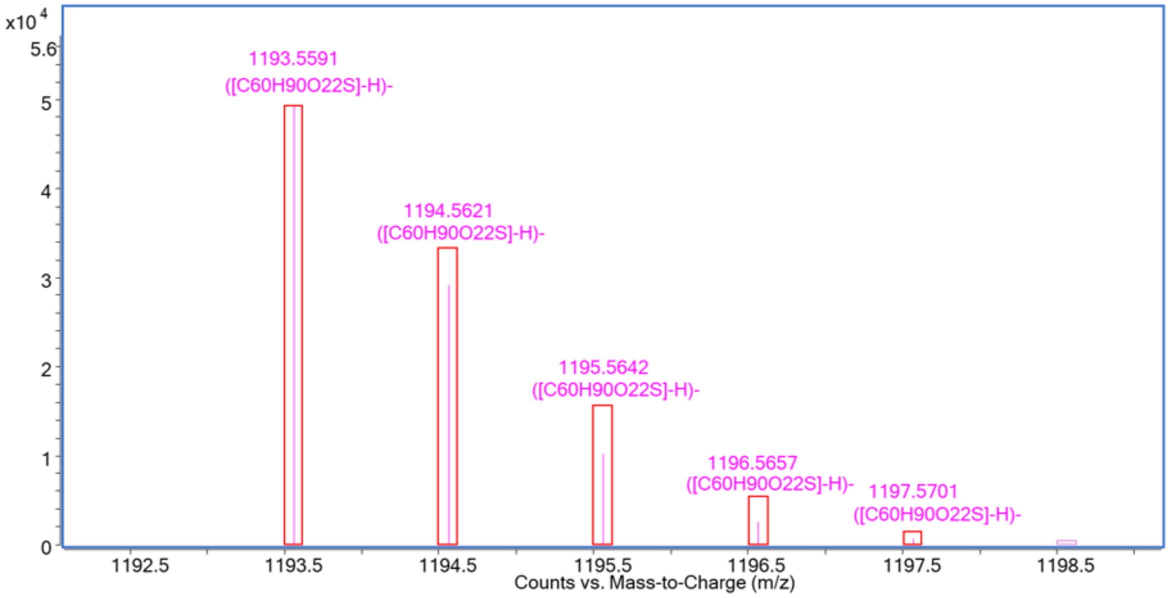

Figure S6. ESI- LC-HRMS full scan analysis of putative gambieroxide detected in *G. australes* at 5.32 min.

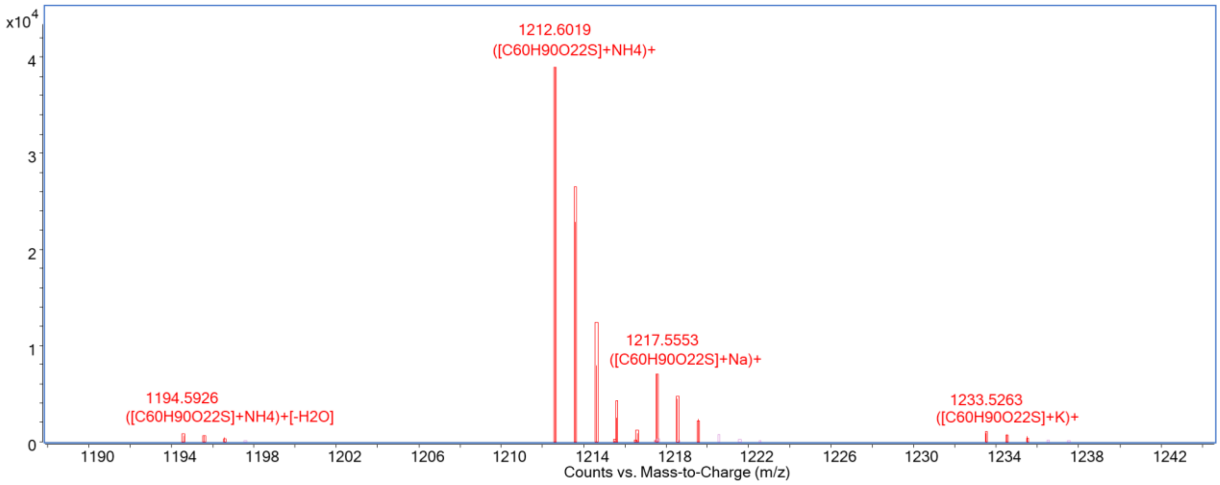

Figure S7. ESI+ LC-HRMS full scan analysis of putative gambieroxide detected in *G. australes* at 5.32 min.

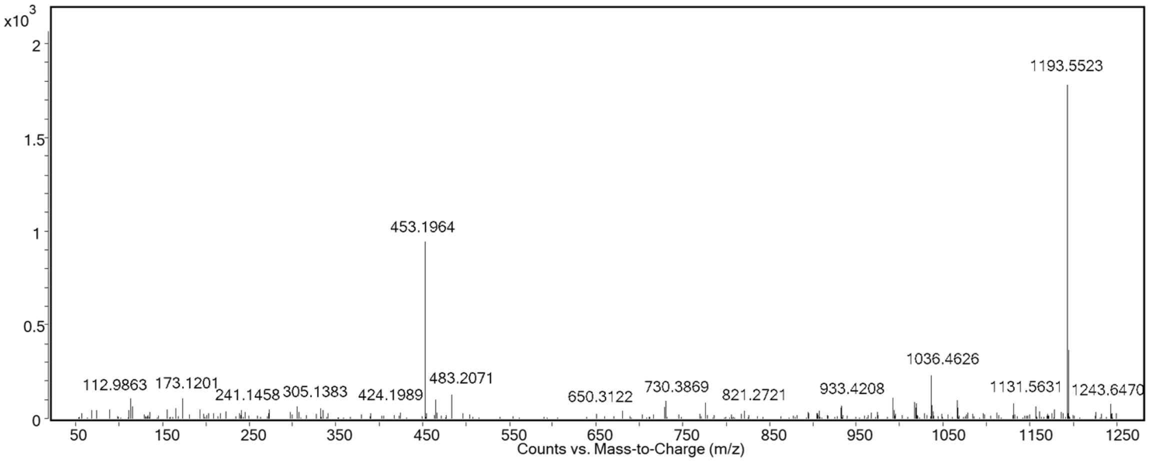

Figure S8. ESI- targeted HRMS/MS spectrum of putative gambieroxide selecting [M-H]- ion in *G. australes* at a collision energy of 50 eV.

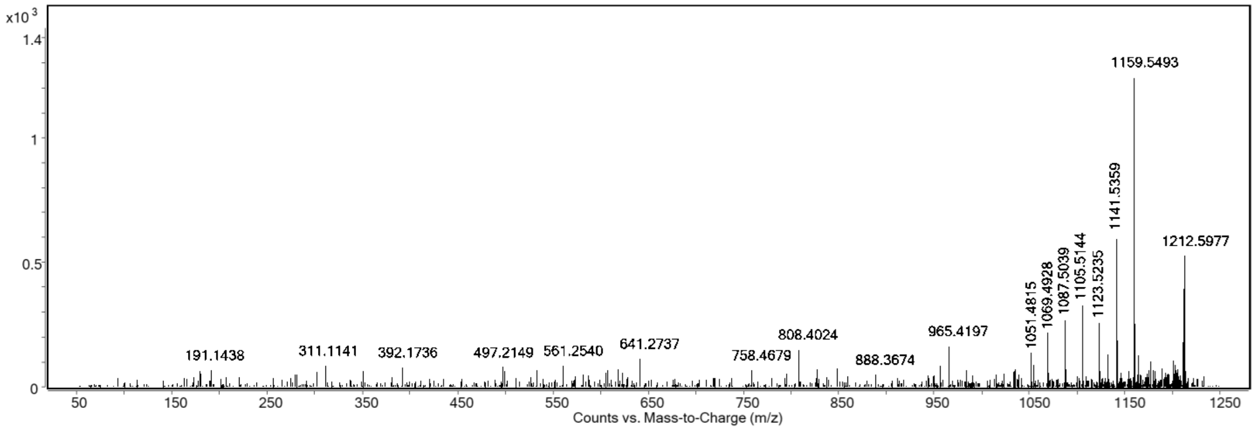

Figure S9. ESI$^+$ Targeted HRMS/MS spectrum of putative gambieroxide selecting $[M+H]^+$ ion in *G. australes* at a collision energy of 20 eV.

Table S6. Accurate mass measurements using LC-HRMS full scan analysis in ESI⁻ and ESI⁺ mode for putative gambieric acid C.

Specie	Sample ID	Injection N°	ESI⁺ Gambieric Acid C Ion $[M+H]^+$	$[M+NH_4]^+$	$[M+Na]^+$	Retention time (min)	ESI⁻ Gambieric Acid C Ion $[M-H]^-$	Retention time (min)
G. australes	IRTA-SMN-17-189	R1	1185.6961 (Δppm = +2.4)	1202.722 (Δppm= +1.9)	1207.6751 (Δppm = 0.0)	8.90	1183.6785 (Δppm = −0.1)	8.90
		R2	1185.6969 (Δppm = +3.1)	1202.7232 (Δppm = +2.9)	1207.6776 (Δppm = +2.1)	8.88	1183.6774 (Δppm = −1.0)	8.88
		R3	1185.6959 (Δppm = +2.3)	1202.7222 (Δppm = +2.1)	1207.6766 (Δppm = +1.2)	8.88	1183.6776 (Δppm = −0.8)	8.88
	IRTA-SMN-17-253	R1	1185.6945 (Δppm = +1.1)	1202.7217 (Δppm = +1.7)	1207.6751 (Δppm = 0.0)	8.89	1183.6774 (Δppm = −1.0)	8.89
		R2	1185.6943 (Δppm = +0.9)	1202.7197 (Δppm = 0.0)	1207.6765 (Δppm = +1.2)	8.89	1183.6778 (Δppm = −0.7)	8.89
		R3	1185.6908 (Δppm = −2.0)	1202.7207 (Δppm = +0.8)	1207.6749 (Δppm = −0.2)	8.89	1183.6769 (Δppm = −1.4)	8.89
	IRTA-SMN-17-244	R1	1185.6944 (Δppm = +1.0)	1202.7215 (Δppm = +1.5)	1207.6762 (Δppm = +0.9)	8.90	1183.6774 (Δppm = −1.0)	8.90
		R2	1185.6940 (Δppm = +0.7)	1202.7202 (Δppm = +0.4)	1207.6766 (Δppm = +1.2)	8.89	1183.6768 (Δppm = −1.5)	8.90
		R3	1185.6945 (Δppm = +1.1)	1202.721 (Δppm = +1.1)	1207.6751 (Δppm = 0.0)	8.89	1183.6771 (Δppm = −1.3)	8.88
	IRTA-SMN-17-162	R1	1185.6875 (Δppm = −4.8)	1202.7193 (Δppm = −0.3)	1207.6779 (Δppm = +2.3)	8.89	1183.6763 (Δppm = −1.9)	8.90
		R2	1185.6875 (Δppm = −4.8)	1202.7188 (Δppm = −0.7)	1207.6751 (Δppm = 0.0)	8.89	1183.6763 (Δppm = −1.9)	8.90
		R3	n.d.	n.d.	n.d.	n.d.	1183.6767 (Δppm = −1.6)	8.88
	IRTA-SMN-17-164	R1	1185.6923 (Δppm = −0.8)	1202.7178 (Δppm = −1.6)	1207.6798 (Δppm = +3.9)	8.89	1183.6764 (Δppm = −1.9)	8.89
		R2	1185.6929 (Δppm = −0.3)	1202.7203 (Δppm = +0.5)	1207.6756 (Δppm = +0.4)	8.89	1183.6757 (Δppm = −2.4)	8.90
		R3	1185.6945 (Δppm = +1.1)	1202.721 (Δppm = +1.1)	1207.6751 (Δppm = 0.0)	8.89	1183.6756 (Δppm = −2.5)	8.88

	IRTA-SMN-17-271	R1	1185.6958 (Δppm = +2.2)	1202.7218 (Δppm = +1.7)	1207.6758 (Δppm = +0.6)	8.90	1183.6782 (Δppm = −0.3)	8.88
		R2	1185.6963 (Δppm = +2.6)	1202.7223 (Δppm = +2.2)	1207.6753 (Δppm = +0.2)	8.88	1183.6778 (Δppm = −0.7)	8.88
		R3	1185.6967 (Δppm = +3.0)	1202.7227 (Δppm = +2.5)	1207.6766 (Δppm = +1.2)	8.89	1183.6780 (Δppm = −0.5)	8.88
G. excentricus	IRTA-SMN-17-407	R1	n.d.	n.d.	n.d.	n.d.	n.d.	n.d.
		R2	n.d.	n.d.	n.d.	n.d.	n.d.	n.d.
		R3	n.d.	n.d.	n.d.	n.d.	n.d.	n.d.
F. paulensis	IRTA-SMN-17-209	R1	n.d.	n.d.	n.d.	n.d.	n.d.	n.d.
		R2	n.d.	n.d.	n.d.	n.d.	n.d.	n.d.
		R3	n.d.	n.d.	n.d.	n.d.	n.d.	n.d.
G. sp 2	0010G-CR-CCAUTH	R1	n.d.	n.d.	n.d.	n.d.	n.d.	n.d.
		R2	n.d.	n.d.	n.d.	n.d.	n.d.	n.d.
		R3	n.d.	n.d.	n.d.	n.d.	n.d.	n.d.

Table S7. Accurate mass measurements using LC-HRMS full scan analysis in ESI⁻ and ESI⁺ mode for putative gambieric acid D.

Specie	Sample ID	Injection N°	ESI⁺ Gambieric Acid D Ion			Retention time (min)	ESI⁻ Gambieric Acid D Ion	Retention time (min)
			$[M+H]^+$	$[M+NH_4]^+$	$[M+Na]^+$		$[M-H]^-$	
G. australes	IRTA-SMN-17-189	R1	1199.7105 (Δppm = +1.4)	1216.7374 (Δppm = +1.6)	1221.6926 (Δppm = +1.5)	8.93	1197.6925 (Δppm = −1.5)	8.93
		R2	1199.7099 (Δppm = +0.9)	1216.7370 (Δppm = +1.3)	1221.6918 (Δppm = +0.8)	8.93	1197.6921 (Δppm = −1.8)	8.93
		R3	1199.7098 (Δppm = +0.8)	1216.7368 (Δppm = +1.2)	1221.6917 (Δppm = +0.7)	8.93	1197.6922 (Δppm = −1.8)	8.92
	IRTA-SMN-17-253	R1	1199.7092 (Δppm = +0.3)	1216.7359 (Δppm = +0.4)	1221.6918 (Δppm = +0.8)	8.93	1197.6920 (Δppm = −1.9)	8.92
		R2	1199.7101 (Δppm = +1.1)	1216.7363 (Δppm = +0.7)	1221.6925 (Δppm = +1.4)	8.93	1197.6924 (Δppm = −1.6)	8.92
		R3	1199.7095 (Δppm = +0.6)	1216.7361 (Δppm = +0.6)	1221.6920 (Δppm = +1.0)	8.93	1197.6920 (Δppm = −1.9)	8.93
	IRTA-SMN-17-244	R1	1199.7092 (Δppm = +0.3)	1216.7359 (Δppm = +0.4)	1221.6918 (Δppm = +0.8)	8.93	1197.6926 (Δppm = −1.4)	8.93

		R2	1199.7101 (Δppm = +1.1)	1216.7363 (Δppm = +0.7)	1221.6925 (Δppm = +1.4)	8.93	1197.6928 (Δppm = −1.3)	8.93
		R3	1199.7095 (Δppm = +0.6)	1216.7361 (Δppm = +0.6)	1221.6920 (Δppm = +1.0)	8.93	1197.6918 (Δppm = −2.1)	8.93
	IRTA-SMN-17-162	R1	1199.7079 (Δppm = −0.8)	1216.7359 (Δppm = +0.4)	1221.6920 (Δppm = +1.0)	8.93	1197.6921 (Δppm = −1.8)	8.93
		R2	1199.7059 (Δppm = −2.4)	1216.7333 (Δppm = −1.7)	1221.6914 (Δppm = +0.5)	8.92	1197.6916 (Δppm = −2.3)	8.93
		R3	1199.7063 (Δppm = −2.1)	1216.7344 (Δppm = −0.8)	1221.6903 (Δppm = −0.4)	8.93	1197.6914 (Δppm = −2.4)	8.93
	IRTA-SMN-17-164	R1	n.d.	n.d.	n.d.	n.d.	1197.6924 (Δppm = −1.6)	8.93
		R2	n.d.	n.d.	n.d.	n.d.	1197.6935 (Δppm = −0.7)	8.93
		R3	n.d.	n.d.	n.d.	n.d.	1197.6924 (Δppm = −1.6)	8.93
	IRTA-SMN-17-271	R1	1199.7103 (Δppm = +1.3)	1216.7370 (Δppm = +1.3)	1121.6922 (Δppm = +1.1)	8.93	1197.6932 (Δppm = −0.9)	8.93
		R2	1199.7110 (Δppm = +1.8)	1216.7376 (Δppm = +1.8)	1221.6923 (Δppm = +1.2)	8.92	1197.6927 (Δppm = −1.3)	8.92
		R3	1199.7104 (Δppm = +1.3)	1216.7375 (Δppm = +1.7)	1221.6929 (Δppm = +1.7)	8.92	1197.6926 (Δppm = −1.4)	8.92
G. excentricus	IRTA-SMN-17-407	R1	n.d.	n.d.	n.d.	n.d.	n.d.	n.d.
		R2	n.d.	n.d.	n.d.	n.d.	n.d.	n.d.
		R3	n.d.	n.d.	n.d.	n.d.	n.d.	n.d.
F. paulensis	IRTA-SMN-17-209	R1	n.d.	n.d.	n.d.	n.d.	n.d.	n.d.
		R2	n.d.	n.d.	n.d.	n.d.	n.d.	n.d.
		R3	n.d.	n.d.	n.d.	n.d.	n.d.	n.d.
G. sp 2	0010G-CR-CCAUTH	R1	n.d.	n.d.	n.d.	n.d.	n.d.	n.d.
		R2	n.d.	n.d.	n.d.	n.d.	n.d.	n.d.
		R3	n.d.	n.d.	n.d.	n.d.	n.d.	n.d.

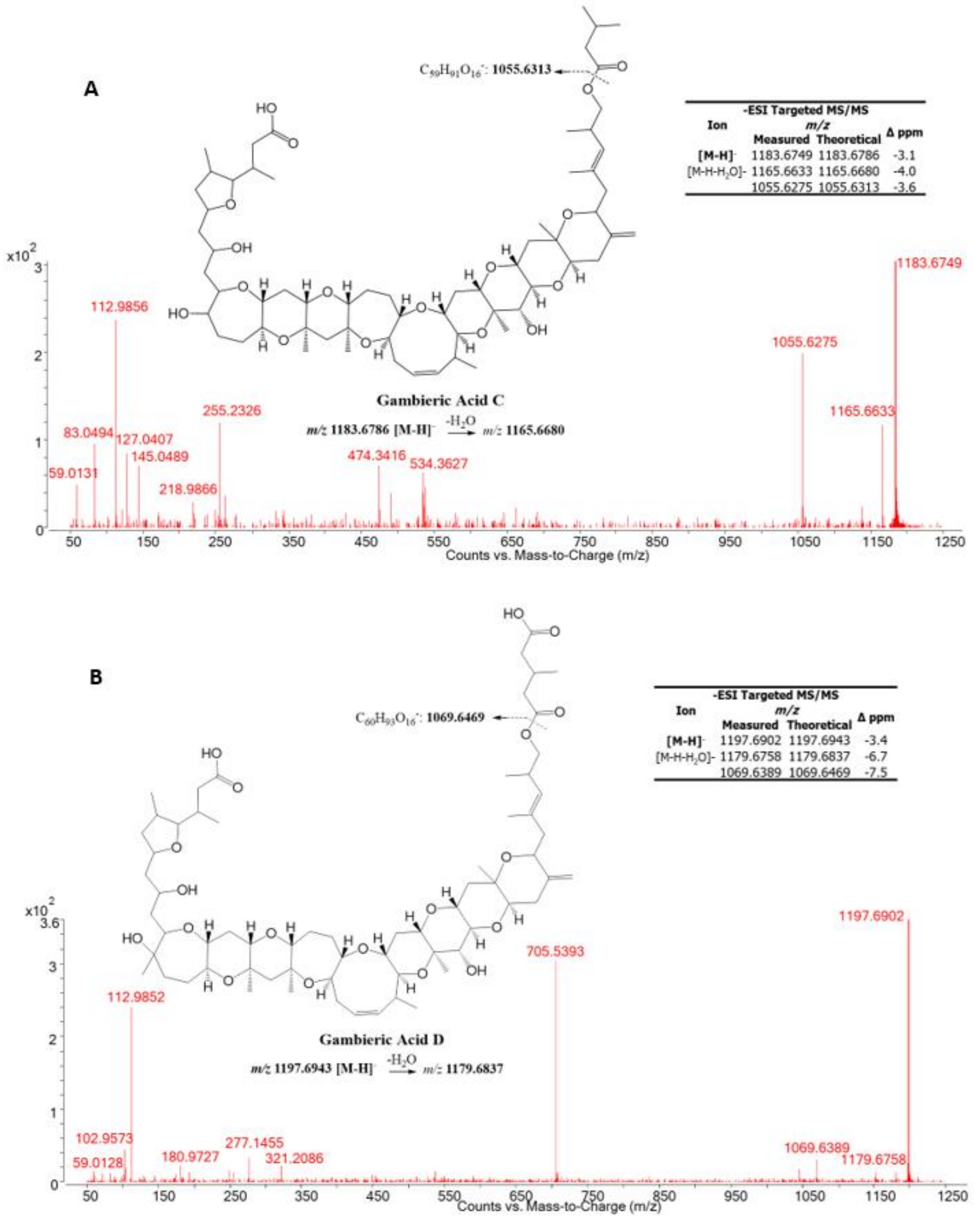

Figure S10. ESI- Targeted MS/MS spectra at an average of 20, 40 and 60 eV of: (**A**) gambieric acid C; (**B**) gambieric acid D detected in *G. australes.*

Table S8. MRM transitions monitored using the LC-MS API 4000 QTrap.

Compound	Retention time (min)*	ESI	MRM Transitions Q1/Q3 (m/z)		CE (ev)	CXP (ev)
MTX1	6.29*	-	$[M-2H]^{2-}/[M-2H]^{2-}$	1689.8/1689.8	−40	−15
			$[M-2H]^{2-}/[HOSO_3]^{-}$	1689.8/96.9	−125	−21
MTX2	Unknown	-	$[M-2H]^{2-}/[HOSO_3]^{-}$	1637.5/96.9	−25	−21
			$[M-3H]^{3-}/[HOSO_3]^{-}$	1091.5/96.9	−125	−21
MTX3	6.19*§	-	$[M-H]^{-}/[M-H]^{-}$	1037.6/1037.6	−40	−15
			$[M-H]^{-}/[HOSO_3]^{-}$	1037.6/96.8	−125	−21
		+	$[M+H]^{+}/[M+H-H_2O]^{+}$	1039.5/1021.5	25	15
			$[M+H]^{+}/[M-SO_3-H_2O+H]^{+}$	1039.5/941.5	35	15
				1039.5/233.1	60	21
				1039.5/109.0	80	21
MTX4	6.08*	-	$[M-2H]^{2-}/[M-2H]^{2-}$	1646.2/1646.2	−40	−15
			$[M-2H]^{2-}/[HOSO_3]^{-}$	1646.2/96.9	−125	−21
desulfo-MTX1	Unknown	-	$[M-2H]^{2-}/[M-2H]^{2-}$	1649.8/1649.8	−40	−15
			$[M-2H]^{2-}/[HOSO_3]^{-}$	1649.8/96.9	−125	−21
didehydro-demethyl-desulfo-MTX1	Unknown	-	$[M-2H]^{2-}/[M-2H]^{2-}$	1641.8/1641.8	−40	−15
			$[M-2H]^{2-}/[HOSO_3]^{-}$	1641.8/96.9	−125	−21
Gambierone	5.94§	-	$[M-H]^{-}/[M-H]^{-}$	1023.5/1023.5	−40	−15
			$[M-H]^{-}/[HOSO_3]^{-}$	1023.6/96.8	−125	−21
		+	$[M+H]^{+}/[M+H-H_2O]^{+}$	1025.5/1007.5	25	15
			$[M+H]^{+}/[M-SO_3-H_2O+H]^{+}$	1025.5/927.5	35	15
				1025.5/219.1	60	21
				1025.5/109.0	80	21

*MTX1 from Wako and samples from a previous study in our laboratory [11] served as reference materials for retention time; § comparison of retention times of gambierone and 44-methyl gambierone (=MTX3) in *G. belizeanus* and *G. australes* served for qualitative identification of these two analogues: since [13] and [14] simultaneously isolated MTX3 independently from *G. belizeanus* and *G. australes*, respectively, the analogue with identical mass and retention time in both species was considered authentic MTX3. A *G. belizeanus* sample from a previous study [11] served as reference material for gambierone.
